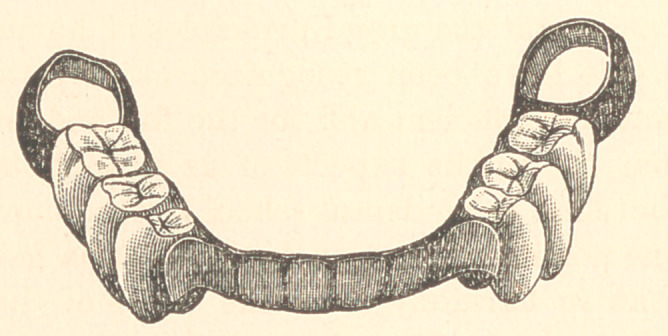# Crown- and Bridge-Work

**Published:** 1892-11

**Authors:** C. M. Richmond

**Affiliations:** New York


### CROWN- AND BRIDGE-WORK.[1]

BY DR. C. M. RICHMOND, NEW YORK.

(Continued from page 740.)

I GIVE in this article a lower bridge (movable) which has been in constant use for eight years, and was the first attempt at this style of work by me. The missing teeth had been lost for some years, and the wisdom teeth had erupted and taken the position of the second molars. They had decayed badly, and were restored with gold crowns before the work was made for the case. After having finished this, a plaster impression was taken of the two gold crowns and the six front teeth; a fusible metal die was cast of each separately. Two bands for the gold molars were then made, using clasp metal No. 30. This works a little harder than twenty-two carat gold, but by getting the bands the right size to begin with, they can be accurately fitted with a small copper-faced hammer, referred to in a former letter. These two bands are then placed in position on the gold crowns previously fastened in position on the two wisdom teeth in the mouth. A plaster impression is then taken of each side of the mouth as far forward as the eye teeth, each side being taken with separate cups. I build up these impressions, after removing them from the mouth, with a little plaster, so that a fusible die can be cast into them without trouble. Each band is then taken off and placed in position in the impression where it belongs, and into the impression I pour fusible metal. This can be done without any danger, as the metal melts at so low a temperature that the gold and metal will not stick together in the slightest degree. After the metal is cold, the impression is taken away and the die is perfect, with the clasp metal band in position, and I have a fac-simile of the mouth with the bands in position. A piece of pure gold plate, No. 30, is now taken and burnished on to the ridge, from the bands forward to the eye teeth. While in this position, the band and gold plate are waxed together at the points of contact. I now pour over the plate and bands some investment material, which, when removed, is really an impression of the die. The plate and bands are placed in position in the impression of investment; this brings them into the same relative situation as when they were on the dies. I now fill with more investment, and, after hard enough, the top is cut through, exposing the point where the gold plates and bands join one another. These

---

[1] Copyrighted, 1892, by Dr. C. M. Richmond.

are placed on the fire, and, when hot, soldered together. As will be seen by referring to the cut, the plate does not touch the gum tissues or come far enough forward to show on the face of the eye teeth. In this case I make the parts separately, like the others I have described, and in the same way, only there is no clasp in front. A piece of pure gold is burnished into the die made of the six front teeth, the same shape as shown in the finished case. I invest this piece, and using a piece of clasp material, which I bend near enough to the shape of the gold it is to cover, so it can be soldered into position without trouble. To facilitate matters, I always cut my gold and clasp to a pattern made of a piece of heavy tin-foil, the same as if a whole gold plate or gold for a partial plate were required. After these pieces are finished to this point, the bands with the gold plates soldered to them are placed in position in the mouth. The plate is tied to the front teeth, so that it will be impossible to disturb it while taking the impression for the last model.

The gold pieces being all in place in the mouth, a plaster impression is taken over all. After letting the plaster harden thoroughly it is removed, and the front piece and the bands with the gold plates are placed in the impression, and into this is poured a model of investment, as I wish to use this to do the last soldering on. The case is placed in an articulator and the teeth ground in, letting the lower ends of the teeth go a little below the gold plate, and perfectly fitting the gums, grinding the first bicuspid to completely cover the gold which comes around the distal surfaces of the eye teeth. The teeth are backed with pure gold, waxed into place, and invested with the model which they are ground on to, as it is made of investment. The whole of the soldering is done now. To fasten the teeth on to the plate, solder the teeth together, and lastly solder the front part to the first bicuspids and plate, and make the case complete in one piece, as shown in the cut. The best result with the minimum amount of material is obtained in this operation, and it is in the mouth, perfect from an artistic point of view.

(To be continued.)

## Figures and Tables

**Figure f1:**